# Isotropic and anisotropic processes influence fine-scale spatial genetic structure of a keystone tropical plant

**DOI:** 10.1093/aobpla/plx076

**Published:** 2018-01-06

**Authors:** Addisie Geremew, Melkamu G Woldemariam, Alemayehu Kefalew, Iris Stiers, Ludwig Triest

**Affiliations:** 1 Department of Biology, Vrije Universiteit Brussel (VUB), Pleinlaan,Brussels, Belgium; 2 Boyce Thompson Institute for Plant Research, Ithaca, NY, USA; 3 Department of Plant Biology and Biodiversity Management, College of Natural Sciences, Addis Ababa University, Addis Ababa, Ethiopia

**Keywords:** Anemochory, anisotropic, fine-scale spatial genetic structure, isotropic, papyrus, parentage analysis

## Abstract

Limited seed or pollen dispersal enhances spatial genetic relatedness between individuals (fine-scale spatial genetic structure, FSGS), which usually decreases as a function of physical distance. However, such isotropic pattern of FSGS may not always occur when spatially asymmetric processes, for instance, wind direction during dispersal, are considered in wind-pollinated and -dispersed plants. This study assessed the pattern of FSGS in the keystone tropical wetland plant *Cyperus papyrus* (papyrus) as a function of these isotropic and anisotropic processes. We tested the hypothesis that the FSGS would be influenced by predominant wind direction during pollen and seed dispersal, as well as by the physical distance between individuals. We genotyped a total of 510 adults and 407 juveniles from three papyrus swamps (Ethiopia) using 15 microsatellite markers. In addition, the contemporary directional dispersal by wind was evaluated by seed release-recapture experiments and complemented with parentage analysis. Adults and juveniles differed in the strength of isotropic FSGS ranging from 0.09 to 0.13 and 0.12 to 0.16, respectively, and this suggests variation in dispersal distance. Anisotropic FSGS was found to be a function of asymmetric wind direction during dispersal/pollination that varied between sites. Historical gene dispersal distance was astoundingly low (<4 m), possibly due to localized seed rain. According to our contemporary dispersal estimates, mean pollen dispersal distances were longer than those of seed dispersal (101 and <55 m, respectively). More than two-thirds of seeds and half of pollen grains were locally dispersed (≤80 m). The difference in historical and contemporary dispersal distance probably resulted from the asymmetric wind direction due to change in vegetation cover in the surrounding matrix. We further concluded that, in addition to wind direction, post-dispersal processes could influence gene dispersal distance inferred from the FSGS.

## Teaser

Propagule dispersal shapes fine-scale spatial genetic structure (FSGS), which is mostly a function of physical distance between individuals. However, such a relation with distance may not occur when asymmetric processes, for instance, wind direction during dispersal, are involved. We combined genetic and seed dispersal data to assess the pattern of FSGS in the keystone species *Cyperus papyrus* as a function of distance and wind direction. Differences in FSGS between adults and juveniles were detected as a function of distance between individuals. Fine-scale spatial genetic structure was also found to be a function of wind direction during dispersal. Our findings further suggest post-dispersal processes could also influence FSGS.

## Introduction

Exploring processes or factors driving the distribution of genotypes (spatial genetic structure) is significant for understanding the evolutionary and ecological dynamics of plant populations and generating baseline information for conservation (Loveless and Hamrick 1984; Hardy *et al.* 2006; Rhodes *et al.* 2014). Processes such as demographic changes, selection, dispersal and genetic drift may influence the spatial distribution of genotypes in space and time (Lima *et al.* 2015; Volis *et al.* 2016). These processes are, in turn, influenced by mating systems and the abiotic factors (e.g. wind speed, wind direction, precipitation and slope) limiting relative pollen and seed dispersal (Kuparinen *et al.* 2009; De Cauwer *et al.* 2010; Born *et al.* 2012; Lara-Romero *et al.* 2016). However, it is hard to discern the degree to which each factor modulates the fine-scale spatial genetic structure (FSGS) (Born *et al.* 2012; He *et al.* 2013), particularly when multiple distinct dispersal vectors such as wind, water, animal and human are acting and mixed reproductive modes (both sexual and asexual) are involved (Volis *et al.* 2010; Pandey and Rajora 2012). Understanding factors shaping the FSGS has an implication to reproductive success of species. Kinship (neighbourhood) has been regarded as a significant mechanism influencing the reproductive success of flowering plants via the regulation of positive density-dependent reproduction (Castilla *et al.* 2016). In clonal plants, it influences reproductive success by affecting the amount and pattern of compatible pollen dispersal and mating opportunities of individual plants (Charpentier 2002; Setsuko *et al.* 2013; Binks *et al.* 2015).

Dispersal is the key process linking the spatial pattern of the parent plants and their descendants. Multiple agents contribute to the dispersal of propagules (pollen and seeds) of different plant species (Higgins *et al.* 2003) and of a given species across contrasting spatial scales (Thomson *et al.* 2010; Tamme *et al.* 2014). Wind is one of the agents renown to disperse the propagules of many plants species (Nathan *et al.* 2011; Born *et al.* 2012). Many wetland species have small seeds and distinctive adaptations that facilitate long-distance dispersal by wind (Drezner *et al.* 2001). For example, the efficacy of seed dispersal by wind to wetlands that are not well connected by water flow and to isolated terrestrial wetland types varied from 9 to 50 % and 45 to 50 %, respectively, in terms of proportion of species adaptations to facilitate wind dispersal (Soon 2006). Although dispersal by wind (anemochory) is often a primary stage enabling seeds to enter water (Broadhurst 2015), in wetland plants much emphasis has been given to dispersal by water (Soons 2006). Unlike water, wind can transport propagules in all directions and is, therefore, important for dispersal to upstream wetlands and to wetlands not connected by surface water flows.

The proportion how far wind disperses pollen over seeds determines within population FSGS and the degree of gene flow among populations (Resende *et al.* 2011; Buzatti *et al.* 2012). Two contrasting scenarios of FSGS occur when gene flow is constrained due to limited dispersal of pollen over seed and vice versa: (i) if pollen is dispersed over a short distance but seeds are widely and independently dispersed, then weak FSGS would be expected (Resende *et al.* 2011); and (ii) if pollen is extensively dispersed but seed dispersal is locally restricted, then kinship among individuals will decrease with spatial distance leading to marked FSGS under the isotropic pattern following an isolation-by-distance (IBD) model (Wright 1943; Kalisz *et al.* 2001; Born *et al.* 2012; Buzatti *et al.* 2012).

The IBD model (isotropic approach) explained the spatial pattern of genetic structure in several wetland clonal plants (Paul *et al.* 2011; Triest *et al.* 2014; Ahee *et al.* 2015). Nevertheless, this model only considers the Euclidean distance between individuals/populations and assumes homogeneity in landscape or environmental variables, which is not the case for most natural systems (Born *et al.* 2012; He *et al.* 2013). In addition to the breeding system and effective plant density, microenvironmental variation can influence pollen and seed dispersal distances with profound effects on FSGS (Vekemans and Hardy 2004; Born *et al.* 2012; He *et al.* 2013). In such instances, it is imperative to understand how spatially asymmetric processes like wind direction and wind speed during dispersal potentially shape the FSGS of wind-pollinated and -dispersed species. For example, wind-driven dispersal has resulted in directional asymmetry in kinship (anisotropy) between individuals (Bullock and Clarke 2000; Born *et al.* 2012). The assumption here is that the dominant wind might enhance kinship between individuals along the major wind axis, which should result in a slow decline of kinship with distance.

The combined approach (isotropic and anisotropic) has been applied to investigate FSGS patterns in wind-pollinated terrestrial plants (Austerlitz *et al.* 2007; Born *et al.* 2012), wind-dispersed fungal spores (Rieux *et al.* 2014) and direction of human genetic differentiation (Jay *et al.* 2013). The association between distance and FSGS (isotropic pattern) of wind-pollinated and - dispersed emergent aquatic plants has been well established (Triest *et al.* 2014; Ahee *et al.* 2015); however, the effect of wind dispersal direction (anisotropic pattern) is unknown. To understand such patterns, it is essential to compare FSGS across different life stages to determine the relative influences of various factors and selection pressures (Benard and McCauley 2008) and to infer the demographic dynamics and persistence (Kalisz *et al.* 2001; Jacquemyn *et al.* 2006). Different factors may act differently on the FSGS across various life stages starting from recruitment, particularly in perennial plants (Li *et al.* 2005; Sarneel *et al.* 2014). In addition to the FSGS, a parentage analysis provides a direct estimate of the gene flow resulting from pollen and seed dispersal. However, the application of this direct method to investigate pollen and seed dispersal in wetland clonal emergent macrophytes is still limited, possibly due to the difficulty in sampling different cohorts *in situ* in hydrodynamic wetland systems with a muddy mat.

In this study, we assessed the patterns of FSGS in adult and juvenile populations of a keystone, wind-pollinated and dispersed macrophytes, *Cyperus papyrus* (papyrus) genotyped using 15 microsatellite markers. *Cyperus papyrus* is native to wetlands in tropical Africa (Terer *et al.* 2012). Seedling recruitment in papyrus populations has been studied (Terer *et al.* 2015); however, the pattern in FSGS along its primary and secondary dispersal agents, wind and water, respectively, is still unknown. In addition, the contemporary directional dispersal by wind was evaluated by seed release-recapture experiments and complemented with parentage analysis. We hypothesized that seed and pollen dispersal would be strongly dictated by anisotropic (the predominant wind direction) as well as isotropic (the physical distance between individuals) processes, thus resulting in a FSGS difference between adults and juveniles. We asked the following questions: (i) is there a difference in isotropic FSGS between adults and juveniles of a *C. papyrus* population? (ii) do *C. papyrus* populations demonstrate an anisotropic pattern of FSGS along a predominant wind direction? (iii) is contemporary seed dispersal influenced by wind direction? and (iv) can realized gene flow within the range of the indirect method (FSGS) be inferred from direct methods (parentage analysis and seed dispersal experiments)?

## Methods

### The study area and sampling sites

**Figure 1.**
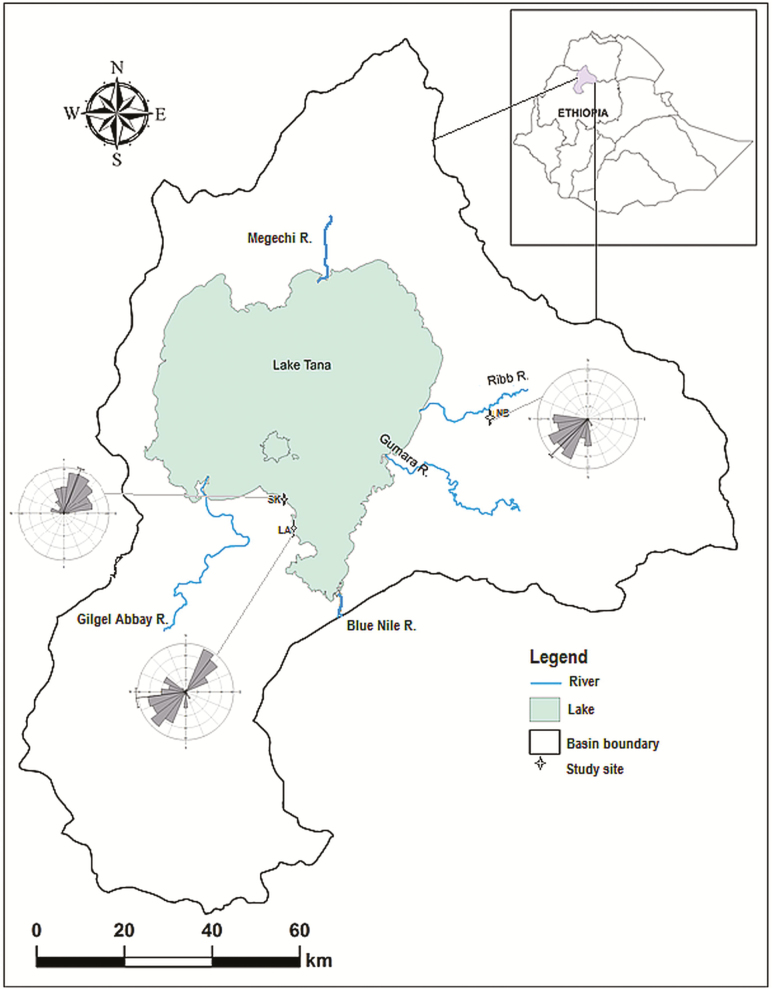
Sampling locations in Lake Tana basin. The three sites (SK, NB and LA) at which *Cyperus papyrus* was sampled in the basin are indicated. The site abbreviations are explained in Table 1. The wind-roses summarize wind direction frequency measured in three closest weather stations from 1985 to 2010 during the flowering and seed dispersal period (June to November).

The present study was carried out in three papyrus swamps located in the Lake Tana basin, Ethiopia, from June to November 2015 (Fig. 1) **[see Supporting Information—Table S1; Photo S1 & S2]**. The climate of the area is classified as tropical highland monsoon where the distribution of rainfall is gauged by the shift of the inter-tropical convergence zone. Various land use matrices such as forest land, shrub land, grass land, cultivated fields and wetlands with pockets of papyrus vegetation fringe the shoreline (Friis *et al.* 2010; Minale and Rao 2012; Sewnet 2015; Hassen and Assen 2017) **[see Supporting Information—Table S1]**. The lake surface flow and recirculation are thought to be regulated by the inflow and predominant wind direction (Dargahi and Setegn 2011). Our study populations, on the east and west sides of Lake Tana, Ethiopia, are situated in regions that exhibit predominant north-east to south-west and west to north-east wind directions, respectively (Dargahi and Setegn 2011). Three papyrus swamps, namely Sekelet Georgis (SK), Ambo-Bahir (LA) and Nabega (NB) (Table 1), were selected bearing, exposure to major winds either from south-west or the north-east, a complex surrounding landscape matrix, and a small swamp area (<5 ha). SK and LA are swamps located in south-western Tana and embedded in the shoreline, whereas NB is situated in eastern Tana and quite isolated from Lake Tana. Although SK and LA are hydrologically connected on the lakeside, particularly during the rainy season, these swamps are separated by a strip of woodland and agricultural landscape on the land side. Although the recruitment history of papyrus in these swamps is unknown, different age groups form a mosaic pattern (personal field observation). Due to the plain topography on the highlands and low vegetation cover, these swamps are free of any barrier that could hinder dispersal by wind.

**Table 1.** Isotropic and anisotropic FSGS estimates of three papyrus swamps in the Lake Tana basin (Ethiopia). *G*: number of multilocus genotypes; *F*_1_: kinship coefficient for the first distance class; *b*(log): regression slope for isotropic analysis; *Sp*: intensity of spatial genetic structure; *Nb*: neighbourhood size; *σ*_g_: gene dispersal distance; *S*_r_: selfing rate; *b*log_Max_ and *b*log_Min_: regression slope at the maximum and minimum bearings; and F1_Max_ and F1_Min_: kinship coefficients for the maximum and minimum bearings of correlations. ns: not significant and * significant at *P* < 0.05. The anisotropic analysis was applied only for the adults and juveniles pooled for each population.

Sites	Stage	Isotropic							Anisotropic							
									Maximum correlation				Minimum correlation			
		*G*	*F*_1_	*b*(log)	*Sp*	*Nb*	*σ*_g_	*S*_r_	*θ*_Max_	*b*log_Max_	F1_Max_	*Sp*_Max_	*θ*_Min_	*b*log_Min_	F1_Min_	*Sp*_Min_
Nabega (NB)	Adult	206	0.138*	−0.076*	0.09	11.42	3.03	0.002^ns^	170	−0.011	−0.003	0.011	233	−0.049*	−0.031*	0.048
	Juvenile	116	0.172*	−0.082*	0.16	6.35	2.25	0.083*	–	–	–	–	–	–	–	–
Ambo- Bahir (LA)	Adult	168	0.144*	−0.076*	0.09	11.23	3.52	0.043^ns^	190.3	0.005	0.034	−0.005	39.01	−0.001*	−0.003*	0.001
	Juvenile	139	0.172*	−0.096*	0.12	8.64	2.33	0.000	–	–	–	–	–	–	–	–
Sekelet (SK)	Adult	136	0.205*	−0.079^ns^	0.13	10.01	2.06	0.007^ns^	290	−0.021	−0.002	0.021	60	−0.131*	0.026*	0.106
	Juvenile	152	0.242*	−0.109*	0.15	4.95	1.98	0.000	–	–	–	–	–	–	–	–

### Seed dispersal experiment

Mean monthly wind directions when *C. papyrus* frequently flowers and disperses (June to November) for the last 25 years (1985 to 2010) were taken from the nearest weather stations. We applied a Weibull likelihood (LOD) density function to complete missing wind data (Bekele and Palm 2009). Direct and indirect methods were used to infer seed and pollen dispersal (cumulative gene dispersal). The FSGS analysis was used as an indirect approach of estimating gene dispersal. The direct method was based on measurements of dispersal distances by a release-recapture experiment (Bullock *et al.* 2006) and parentage analysis. To test for contemporary dispersal by wind, five individuals (total of 30 individuals for 6 days) of *C. papyrus* representing different heights and 20 m apart were selected at each site. The shoots (umbels) of these individuals were partially covered with a cotton glued on the lower surface. A total of 9000 nutlets were painted with five glowing colours: red, white, pink, blue and yellow (*n* = 1800 per colour). Following Sarneel *et al.* (2014), 100 nutlets were placed (not glued) on the cotton-covered individuals (*n* = 100 per individual per colour per day) to mimic spikes before the onset of the windiest hours (2:00 to 4:00 pm). Seeds released were recaptured within a 200 m radius from the source in eight directions (E, NE, N, NW, W, SW, S and SE). We limited the recapturing effort to 85 % of the total released seeds. The direction and distance of each recovered seed dispersed by wind were measured using a handheld compass and a GPS (Garmin GPS 60CSx). We complemented the wind direction data of the last 25 years with the release-recapture experiment to account for possible heterogeneous distribution of juveniles relative to adults while sampling for FSGS and parentage analysis.

### Plant tissue sampling

A total of 960 *C. papyrus* individuals were sampled for genotypic analysis. At each swamp, papyrus shoots were collected from adults along two parallel transects (45 per transect) established based on wind direction. In addition, 30 (adults) and 60 (juveniles) were systematically sampled up to 10 m away from either of the transects **[see Supporting Information—Figure S1]**. We applied this sampling design to optimize the spread distance and the angle between the pairs of individuals for anisotropy FSGS and parentage analyses. Pending genomic DNA extraction, shoots were kept dry on silica gel.

### DNA extraction and genotyping

Genomic DNA was extracted from dried leaves of adults and juveniles using the commercial E.Z.N. A® *SP* plant DNA Miniprep Kit (Omega Bio-Tek, Inc.). Fifteen microsatellite primers developed for *C. papyrus* (Triest *et al.* 2013; Geremew *et al*. under review) were used to genotype. Conditions for PCR amplification were as stated in Triest *et al.* (2013). Amplicons were separated using ABI 3730xl genetic analyzer (Applied Biosystems) and fragment lengths (allele sizes) were estimated relative to the internal size standard (LIZ500). We scored allele sizes manually for each microsatellite locus per individual with GeneMarker® v2.4.1 (SoftGenetics LLC, State College, PA, USA).

### Statistical analysis

Thirty-three individuals showing missing values for more than seven microsatellite loci after amplifying and genotyping two times were discarded from the analysis. We applied a multilocus match function in GenAlEx v. 6.5 (Peakall and Smouse 2006) to identify individuals with identical multilocus genotypes (MLGs). Clonal duplicates (total = 20; 1–4 duplicates per swamp) were removed after estimating the probability of not being separated from each other by a sexual event using GenClone 2.0 (Arnaud-Haond and Belkhir 2007). The subsequent analyses used 917 individuals with distinct MLGs (hereafter referred to as genets) for both adults and juveniles. The frequency of null alleles and a significant departure from Hardy–Weinberg equilibrium were tested for each locus and population using CERVUS version 3.0.3 (Kalinowski *et al.* 2007).

To examine the kinship between individuals and FSGS (isotropic pattern) within each papyrus swamp, we calculated the spatial autocorrelation over mean multilocus kinship coefficients (*F*_*ij*_) (Loiselle *et al.* 1995) at genet level. This coefficient allows to use genotype data depending on a combination of multiple polymorphic loci and is comparatively less biased while alleles with low frequency are considered. To estimate the average *F*_*ij*_, we selected 10 distance classes of 5, 10, 50, 100, 150, 200, 250, 350, 400 and 450 m with even numbers of comparisons between individual pairs in each distance interval (100 per interval). The *F*_*ij*_ was plotted against the distance classes and 95 % confidence intervals generated following 100000 permutations for genets with and without resampling methods (Alberto *et al.* 2005). The strength of the FSGS (*Sp*; Vekemans and Hardy 2004) was computed as *Sp* = *b*/(*F*_(*i*)_ – 1), where *F*_(*i*)_ is the kinship coefficient for the first distance interval, and *b* is the linear slope of the autocorrelogram. Neighbourhood size (*Nb*) as *Nb* = 4*πD*_e_*σ*^2^ or *Nb* = 1/*Sp*, gene dispersal distance (*σ*_g_) and selfing rate (*S*_r_) were estimated at an effective population density (*D*_e_) of 0.1 m^2^ calculated as one-tenth of the observed density in the field. One-tenth of the census density has been recommended as an average correction factor to adjust the effective population density used for simulating *Nb* (Frankham 1995). All these isotropic FSGS analyses were run by SPAGeDi 1.5a (Hardy and Vekemans 2002).

We assessed directional patterns in FSGS using anisotropic analysis (Rosenberg 2000). Directional spatial autocorrelation helps to explore factors modulating genetic structure (Rosenberg 2000). Bearing analysis estimates the direction of strongest correlation between a genetic data matrix generated from kinship coefficient values (*F*_*ig*_) and a spatial distance matrix. This method employs a Mantel test to associate a kinship matrix with a transformed distance matrix generated for a set of wind directions, measured as angles. As pollination and seed dispersal would both be strongly affected by wind direction, we hypothesized that the slope of the regression between kinship coefficient values (*F*_*ij*_) and the spatial distance between individuals would be less negative along the wind vector than across it. We expected that along the wind vector, the stronger the wind, the closer the regression slope would approximate to zero. Using PASSAGE 2 (Rosenberg and Anderson 2011) we were able to find the bearing angle denoting the strongest (*θ*_Min_, minimal Mantel correlation) and weakest FSGS directions (*θ*_Max_, maximal Mantel correlation). The significance of the correlations was obtained using a permutation test (999 permutations). We calculated the intensity of FSGS *θ*_Min_ and *θ*_Max_ for the pooled data sets of adults and juveniles of each population. Circular histograms and calculation of the circular mean for wind directions and angles were produced using the software Oriana version 2.02e (Anglesey, Wales). The significance of directionality in the contemporary seed dispersal by wind was tested with Rayleigh’s test of uniformity in the same software.

The exclusionary power of each locus was tested prior to parentage analysis. Parentage analysis was carried out for juveniles where both parents were unknown by applying the maximum LOD method (Marshall *et al.* 1998) implemented in CERVUS version 3.0.3 (Kalinowski *et al.* 2007). We used the LOD score to detect the most probable parent–offspring pairs, parent pairs, and single parents. Since critical delta is not meaningful for single parents in parent pair analysis with unknown sexes, we only limited to the critical LOD scores. Confidence intervals (both relaxed, 85 % and strict, 95 %) during parentage assignment were estimated based on simulations of parents (adults) and offspring (juveniles) genotype allele frequencies at 10000 mating events; zero genotyping error; 15 minimum number of loci and 0.75 as a proportion of candidate parents sampled. The potential pollen donor and seed parent of juveniles were identified by simple exclusion based on MLGs for all adults of each swamp. This method accounts mismatching between offspring and parents genotypes to nullify parent–offspring hypotheses. During the assignment, if only a single adult is assigned, it is assumed to be the seed parent. We assumed secondary dispersal when juveniles could not be assigned to the presumed parents. When we identified a juvenile comprised of a parent pair within a site, the closest parent was considered to be the mother plant (Dow and Ashley 1996). Such strategy has been indicated as a conservative estimate of seed dispersal distances (Martins *et al.* 2012) and applied for monoecious species in combination with preceding knowledge of pollen and seed dispersal vectors (Sebbenn *et al.* 2010). Pollen and seed dispersal curves were calculated using the distance between the two parents of a given juvenile, and the distance between the juvenile and adult, respectively.

## Results

### Isotropic pattern of FSGS

**Figure 2.**
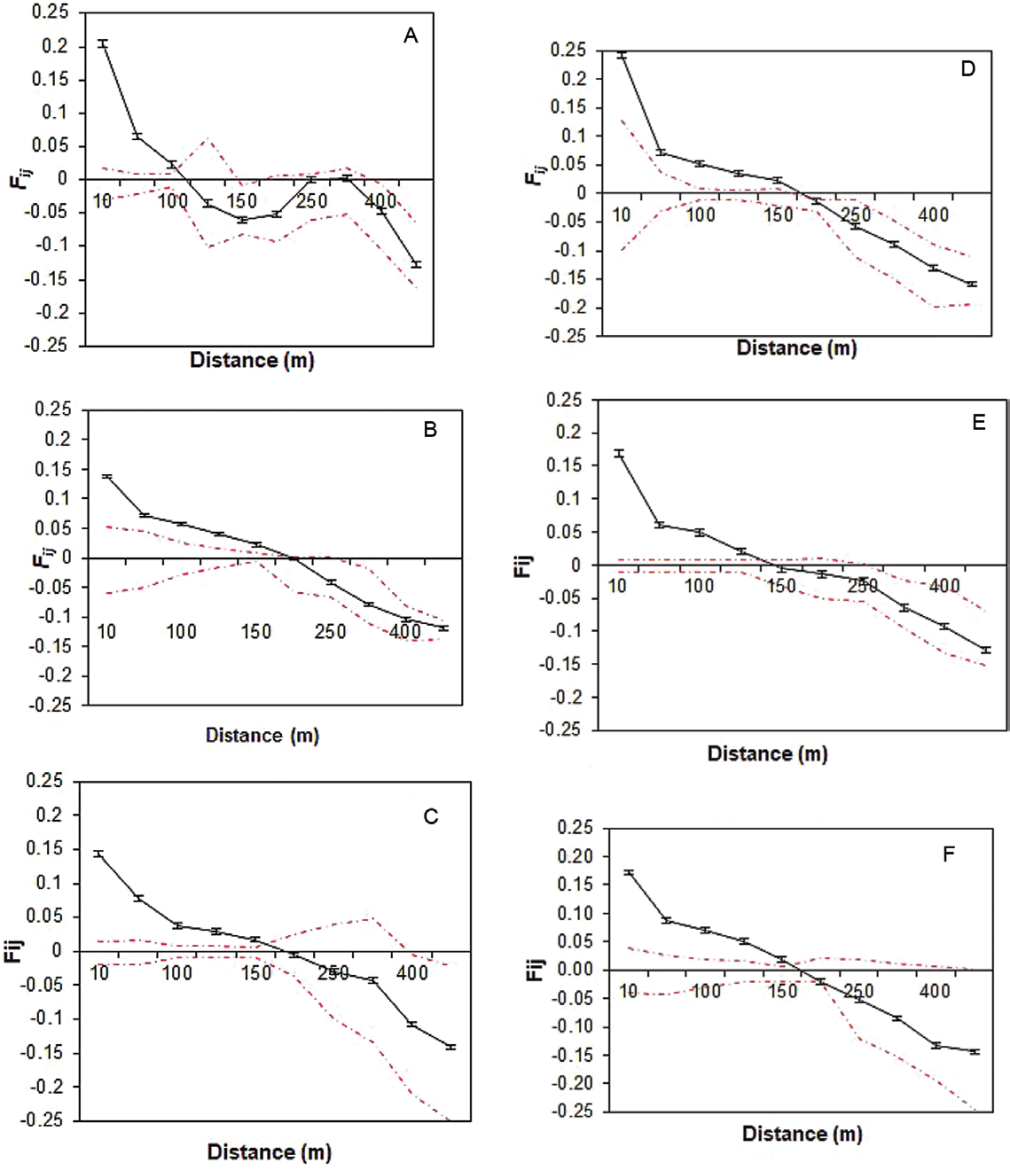
Correlograms of kinship coefficients (*F*_*ij*_) over 10 distance intervals for papyrus adults (A–C) and juveniles (D–F) in SK, NB and LA swamps, respectively (abbreviations in Table 1). Bars represent standard error. The dashed lines represent the upper and lower 95 % confidence limits.

At each of the papyrus swamps (SK, NB and LA), isotropic FSGS for adults and juveniles revealed that kinship (*F*_*ij*_) between individuals decreased with spatial distance (Fig. 2A–F). In both adults and juveniles, strong significant positive values of *F*_*ij*_ were found for distances less than 162 and 150 m, respectively. Beyond these distances, the kinship coefficient decreased within the 95 % confidence intervals. Overall, in each swamp, the juveniles showed relatively higher *F*_*ij*_ compared to the adults (Table 1). The FSGS was stronger in juveniles of NB and SK with *Sp* values of 0.16 and 0.15, respectively, than in adult populations of LA, SK and NB (*Sp* = 0.1). The regression slopes were significant, except for the adults in SK (Table 1). Permutation tests for an equal number of pairs of comparisons resulted in converged Wright’s neighbourhood sizes (*Nb*) and effective gene dispersal distances (*σ*_g_) in adult and juvenile individuals per swamp. While *Nb* values ranged from 5 (SK juveniles) to 11 (NB adults), indirect measures of *σ*_g_ varied from 1.98 m (SK juveniles) to 3.52 m (LA adults). Except for the significant selfing rate (*S*_r_) detected in NB juvenile populations, overall, *S*_r_ was not significantly different from zero. *S*_r_ spanned from zero to 0.083.

### Anisotropic pattern of FSGS

**Figure 3.**
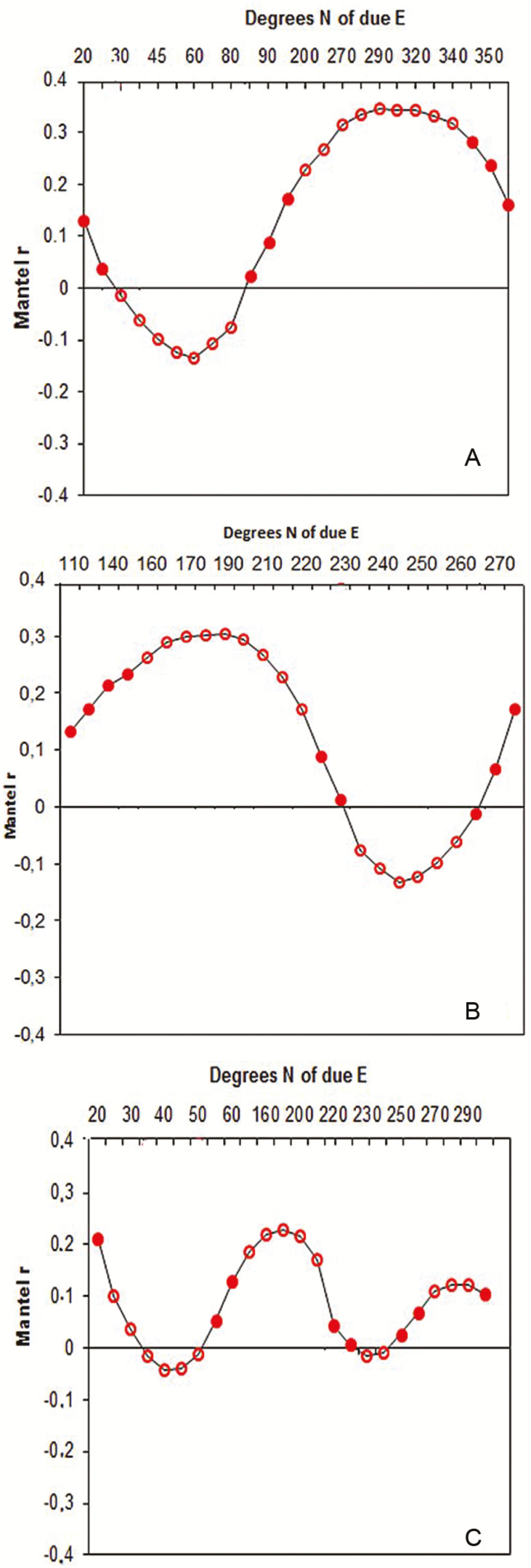
Bearing correlograms generated for wind directions. For a series of wind direction, the Mantel correlation coefficient between genetic relatedness for papyrus individuals and transformed distance matrix plotted for (A) Sekelet (SK), (B) Nabega (NB) and (C) Ambo-Bahir (LA). Significance after permutations represented by open circles.

To explore whether the pattern in FSGS was either explained by only distance or by wind direction that could potentially impact pollen and/or seed dispersal, we applied bearing analysis based on current and wind data over the last 25 years. Anisotropic spatial autocorrelation for a total of 24 fixed bearings revealed significant associations between pairwise kinship metrics and spatial distance at different initial minimal *θ*-values north from due east (Fig. 3A–C). The directions of significant maximum positive correlations were recorded at 190.3° (NE–SW), 290.4° (SE–NW) and 170° (NW–SE) for LA (*r* = 0.228), SK (*r* = 0.345) and NB (*r* = 0.304), respectively. In contrast, we found the strong negative minimal correlation at 40° (SW–NE), 60° (SW–NE) and 233° (NE–SW) for LA (*r* = −0.134), SK (*r* = −0.041) and NB (*r* = −0.138), respectively. The regression slopes for the minimum bearing were significantly negative for LA (*b*log_Min_ = −0.001), NB (*b*log_Min_ = −0.049) and SK ( *b*log_Min_ = −0.131), indicating dispersal is strongly directional and influences the FSGS in each swamp (Table 1).

The wind patterns analysed using circular statistics over the last 25 years were significantly related to the south-west in NB (mean = 225.065°, Rayleigh test, *P* < 0.01) and to the north-east in SK (mean = 20.497°, *P* < 0.01; Table 2) which corroborated the bearing where maximum correlation occurred (Fig. 3A and B). Similarly, the oscillating pattern (Fig. 3C) where peaks of maximum correlation were attained in LA matched the pattern observed in the 25-year wind data (Fig. 1, rose diagrams; Table 2). In this swamp, wind showed a directionality to the south-west and to the north-east, despite the strength clined to the south-west as also seen in the correlation analysis (Fig. 3C).

**Table 2.** Mean seed dispersal distance by wind and directions within a radius of 200 m from the source point, ‘mother plant’ in three papyrus swamps. Statistical significance of dispersal direction in terms of degree was tested using Rayleigh test (*p*). * Significant at *P* < 0.05, ** at *P* < 0.001 and ns: not significant. Values in the bracket represent the standard error of the means seed dispersal distance in each direction. Note that the means were calculated for the number of observations recorded.

Sites	Variables	Seed dispersal directions								Direction (1985–2010)
		E	NE	N	NW	W	SW	S	SE	
NB	# Observation	8	6	20	68	528	1422	196	116	218
	Mean angle (°)	90^ns^	7.3^ns^	1.0^ns^	282.5*	272.1**	223.3**	179.6**	145.7*	225.07**
	Mean distance (m)	16.3 (2.5)	25 (4.2)	8.5 (1.7)	17.9 (2.1)	31.3 (5.3)	65.8 (8.5)	32.9 (4.8)	4.8 (1.6)	–
SK	# Observation	109	1817	842	140	39	10	7	9	618
	Mean angle (°)	87.3**	24.3**	359.4*	290.4*	273.5^ns^	235.2^ns^	179.7^ns^	123.4^ns^	20.50**
	Mean distance (m)	20.1 (3.4)	27.1 (2.7)	22.5 (4.6)	30.5 (3.1)	40.2 (6.1)	18.6 (2.6)	14.2 (5.2)	19 (5.1)	–
LA	# Observation	11	1735	14	46	109	866	90	21	620
	Mean angle (°)	72.31^ns^	44.53**	360^ns^	303.7*	270**	259.7**	181.2*	136.1^ns^	262.82*
	Mean distance (m)	13.9 (1.7)	28.3 (2.2)	6.9 (3.1)	19.9 (5.3)	20.1 (6.6)	142.3 (9)	21.3 (3.4)	14.6 (3.2)	–

### Contemporary seed dispersal

Of all the seeds experimentally released, 85, 93 and 95 % were recovered for the swamps NB, LA and SK, respectively **[see Supporting Information—Table S2]**. The reconstructed contemporary wind dispersal distance distributions were leptokurtic, with the majority of seeds (94 %) dispersed by wind less than 50 m from the source **[see Supporting Information—Figure S2]**. Dispersal direction showed a unimodal distribution in NB and SK swamps, but a bimodal distribution in LA towards the NE and SW (Table 2) **[see Supporting Information—Figure S1]**. Overall, directional seed dispersal by wind was observed across the swamps studied. The Rayleigh test (*P* < 0.01) demonstrated that within 200 m from the parent plant, the strongest and most significant direction correlated with the longest dispersal distance occurred at 223.3° (SW), 24.3° (NE) and 259.7° (SW) in NB, SK and LA in that order (Table 2). The mean distance over which seeds were dispersed by wind across the eight major direction groups differed within each swamp (Table 2).

### Parentage analysis of recent juveniles

The 15 microsatellite markers employed in this study revealed a high exclusion probability of greater than 99 % for the first parent, the second parent and the parent pair (Table 3) **[see Supporting Information—Table S3]**. This suggested that the 15 microsatellite markers were suitable for parentage analysis. In addition, the overall null allele frequency was low and was not significantly different from zero. A simple exclusion test using both relaxed (85 %) and restricted (95 %) confidence limits showed that the number of juveniles to which parent pairs and single parents were assigned varied among swamps **[see Supporting Information—Figure S3]**. The critical LOD scores for simulation were high (12.3 to 14.7) for the 95 % confidence level. The assignment rates were 3–22 % for the strict (95 %) confidence level and 4–32 % for the relaxed (85 %) confidence level (Table 3). For example, in SK, of the 152 juveniles, 46 (30 %) had matching pair parents and 97 (64 %) were assigned to only one parent within the swamp. In this population, there was no parent pair assigned to the remaining 9 (6 %) juveniles, indicating gene flow from outside the swamp. The assignment results for the remaining two swamps are given in **Supporting Information—Figure S3**.

**Table 3.** Gene diversity, pollen and seed dispersal distances inferred from parentage analysis of papyrus populations and discriminating power of the set of microsatellite markers. *H*_E_: expected heterozygosity; *P*_null_: null allele frequency; PE1 and PE2: exclusion probability for the first and second parent, respectively. Min: minimum and Max: maximum dispersal distance.

Sites	*H*_E_	Exclusion probability				Assignment rate		Parentage analysis					
		*P*_null_	PE1	PE2	PP	Critical LOD (80/95)	Assignment rate (85/95)	Seed (m)			Pollen (m)		
								Min.	Max.	Mean	Min.	Max.	Mean
NB	0.746	0.001	0.992	0.999	1.000	7.5/12.3	29 %/22 %	1.6	160	42.64	4.6	193.7	62.36
LA	0.691	−0.001	0.998	0.999	0.999	9.97/14.73	32 %/16 %	3.4	200	56.55	15.2	200	81.11
SK	0.676	0.002	0.996	0.991	0.999	8.0/13.0	4 %/3 %	2.5	172	34.9	4.7	200	101.2

**Figure 4.**
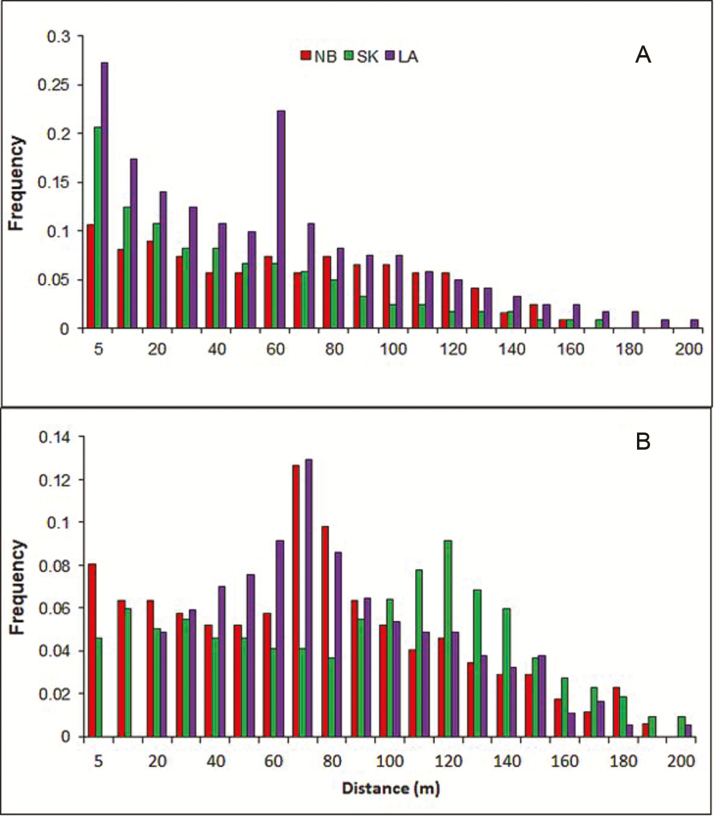
Frequency distribution of distances of seed (A) and pollen (B) dispersals inferred from paternity assignment in the three papyrus swamps: Nabega (NB, red bar), Sekelet (SK, green bar) aJ9nd Ambo-Bahir (LA, purple bar). Linear distance was calculated as mentioned in the Methods section.

The direct estimates of gene flow based on parentage analysis revealed that the observed mean pollen dispersal distance was larger than the seed dispersal distance within each papyrus swamp. About 75 % of the seeds and 42 % of the pollen grains were dispersed ≤80 m (Fig. 4B). The mean seed and pollen dispersal distances ranged, respectively, from 34.5 to 56.55 m and from 62.36 to 101.2 m (Fig. 4; Table 3). Consistent with the release-recapture seed dispersal experiment the maximum seed dispersal distance computed from the parentage analysis (at 95 % confidence) was about 200 m (Fig. 4).

## Discussion

By combining genetic data and dispersal experiments, we assessed isotropic and anisotropic processes influencing the FSGS and seed dispersal and pollen flow patterns in *C. papyrus* populations. Our key findings highlight: (i) significant isotropic FSGS that differed between age groups (adults vs. juveniles), (ii) FSGS of *C. papyrus* is not only a function of distance between individuals, but is also influenced by the asymmetric pattern of wind direction during dispersal (anisotropic pattern), (iii) localized contemporary seed dispersal is driven by wind and (iv) historical gene flow inferred from the FSGS reveals shorter dispersal distance compared to the estimates of contemporary gene flow from parentage analysis and the dispersal experiment.

We found a significant FSGS following an isotropic or IBD pattern within adult and juvenile *C. papyrus* individuals and this could be explained by localized seed or pollen dispersal. Localized seed dispersal enhances spatially dependent recruitment (Chung *et al.* 2011) which results in strong FSGS (Volis *et al.* 2010). However, the effect of seed vs. pollen dispersal on the final FSGS may vary (Resende *et al.* 2011; Freeland *et al.* 2012). In 2D space, significant positive autocorrelations were detected at distances of ≤162 m for adults and ≤152 m for juveniles in the Lake Tana basin (Ethiopia). In a different lake, Lake Naivasha (Kenya), FSGS within *C. papyrus* population has also been detected at about 100 m (Triest *et al.* 2014; Terer *et al.* 2015). The *Sp* statistic is suitable for comparing the intensity of FSGS among species with different mating systems and dispersal modes (Vekemans and Hardy 2004). The observed *Sp* statistics (adults = 0.09 to 0.10 and juveniles = 0.12 to 0.16) were within the range for other wind-pollinated and/or wind-dispersed species (Collevatti *et al.* 2014) and were high compared to species with mixed mating systems and self-incompatible species (Vekemans and Hardy 2004).

In perennial plant species, different cohorts established within a population conceivably resulting in a high genetic diversity, but lower FSGS (Addisalem *et al.* 2016). However, when differences in FSGS between juvenile and adult stages occur, it may reflect disparities between generations in terms of gene flow and demographic processes (Berens *et al.* 2014). The FSGS in *C. papyrus* juveniles was relatively stronger than in adults. Other studies examining FSGS across different stages have either shown a decrease (Zhou and Chen 2010; Chung *et al.* 2011), an increase (Kalisz *et al.* 2001; Jacquemyn *et al.* 2006; Jones and Hubbell 2006; Berens *et al.* 2014) or no change (Berg and Hamrick 1995) in FSGS from juveniles to adult. Several factors may explain the lack of a common pattern encompassing species and site-specific post-dispersal and early selection, besides differences across generations due to different levels of gene flow. The decrease in the strength of FSGS from juveniles to adults could potentially be attributed to post-dispersal thinning (e.g. competition among genets) or selection effects (Hardesty *et al.* 2005; Zhou and Chen 2010). Also, when adult (maternal) plants are at low density which curtails overlap of seed shadows because of germination, juveniles would have stronger FSGS (Chung *et al.* 2011). However, germination rate is low in *C. papyrus* (Boar 2006).

In plants showing vegetative spread and a small number of founders, clonal growth and limited outgoing gene flow, respectively, may also have contributed to the strong FSGS (Hämmerli and Reusch 2003; Pandey and Rajora 2012; Valverde *et al.* 2016). However, our multilocus match analysis revealed only 1–4 individuals with identical MLGs per swamp and was removed from further analysis, so vegetative spread could not explain the observed levels of FSGS. Although selfing has been indicated as one of the factors that reinforces FSGS, our results showed that *S*_r_ was not significant except for NB juveniles. In this population, despite the significant *S*_r_ detected, the stronger kinship between neighbouring juveniles of the same age within population than between juveniles and adults indicates a temporal Wahlund effect owing to localized and directional seed dispersal (Chung *et al.* 2004). Furthermore, the discrepancy in FSGS between juveniles and adults could also be due to microhabitat or microenvironmental heterogeneity and local selection (Zhou and Chen 2010). Pronounced FSGS is expected if neighbouring individuals of a mixture of related parents and progeny require the same microhabitat conditions (Jones and Hubbell 2006).

The wind direction during dispersal reinforces the asymmetric pattern of FSGS in some plant species (e.g. Born *et al.* 2012; Wang *et al.* 2016). Directional dispersal following wind continued over generations results in a predominance of gene flow along or across some axes, and leaves an imprint on FSGS (Born *et al.* 2012). We also observed asymmetric (anisotropic) pattern in FSGS, signifying directionally biased gene dispersal through pollen and seed dispersal along the prevailing wind directions. Consistent with theoretical considerations that wind is a primary mode of both pollination and seed dispersal in *C. papyrus* (Terer *et al.* 2012), the kinship–distance relationship differed periodically along the wind directions in each swamp. In contrast to the present study, Dutech *et al.* (2005) pointed out the lack of strong association between wind dispersal direction and kinship values between individuals of *Quercus lobate* due to wind-dispersed pollen over many kilometres. The minimum bearings where a strong FSGS was observed varied between swamps. Bearings associated with the lowest spatial autocorrelation (ϴ_MIN_) suggest the prominent wind direction at the time of pollen and seed dispersal (Born *et al.* 2012). Significant anisotropic patterns with ϴ_MIN_ = 60° in SK and ϴ_MIN_ = 233° in NB indicate that predominantly south-westerly and north-easterly winds (Dargahi and Setegn 2011), respectively, played a significant role during dispersal and pollination and consequently influenced the pattern of FSGS. In addition, both south-westerly and north-easterly winds influenced seed and pollen dispersal pattern in LA. However, the south-westerly wind was stronger than the north-easterly wind in driving the FSGS pattern. In this swamp specifically, the lowest and F1_Min_ value (−0.003) and the least negative regression slope ( *b*log_Min_ = −0.001) could be associated with these two wind directions, supporting the long-distance pollen and seed dispersal efficacy. Also, based on the field release-recapture experiments the frequency of seed dispersed along the direction of the predominant winds showed an asymmetric pattern. Prevailing and strong winds in a given direction dispersed maximum number of seeds to be dispersed. However, strong winds may be rare events that do not happen every year and in all locations, thus the actual effect of wind on seed dispersal may be stronger than observed in our experiment. It will be important to disentangle whether each of the contemporary pollen and seed dispersals is measurably anisotropic based on the direct estimate.

The short historical gene dispersal distance (range *σ*_g_ = 1.98–3.52) and small neighbourhood size (range *Nb* = 4.95–11.42) observed could be attributed to localized seed dispersal (Soomers *et al.* 2013; Broadhurst 2015). However, the current dominant wind in these swamps could be due to changes in vegetation cover because of extensive agricultural activities (Minale and Rao 2012; Hassen and Assen 2017). Consistent with this assertion our results also indicated that the direct estimate of pollen dispersal distance was indeed nearly double the seed dispersal distance. In monoecious plants, pollen is dispersed over substantial distances (Schuster and Mitton 2000; Pandey and Rajora 2012). De-Lucas *et al.* (2009) have also shown that seed dispersal in wind-pollinated species, rather than pollen flow, is the dominant factor determining the FSGS. In wind-pollinated species, however, an erratic variation of pollen flow may increase mating-pair heterogeneity that would impact the FSGS (Buzatti *et al.* 2012). Additionally, the smallest neighbourhood size (*Nb* = 4.95) and the corresponding shortest gene dispersal distance (*σ*_g_ = 1.98 m) of SK juveniles, for instance, reflect the critical role that genetic drift and inbreeding play in overcoming the effect of gene flow (Fenster *et al.* 2003).

In contrast to the mean seed dispersal estimate from the parentage analysis, the release-recapture experiment showed a relatively long-distance and directional seed dispersal. This discrepancy could be because a parentage analysis may exhibit the pattern of recently recruited generations from the seed bank compared to the release-recapture experiment displaying seed dispersal of up to 200 m in a single generation. In addition, this difference allows us to hypothesize that post-dispersal processes such as seed deposition pattern and recruitment (Vandepitte *et al.* 2009; Segarra-Moragues *et al.* 2016) may contribute to the asymmetric FSGS discussed earlier. The tail of seed dispersal distance that extends the mean value (maximum up to 200 m) also suggests a signature of rare long-distance seed dispersal by wind but with the majority of the seeds being deposited at a short distance from the parent plant. The high number of seeds used and the high recapture effort applied in our experiment indicate that the large proportion of the seeds was recaptured at a short distance from the parent, reflecting localized seed dispersal. Nevertheless, accurate characterization of the tail is enormously difficult (Nathan 2006), especially when plant populations are experiencing substantial immigration from outside populations. For instance, the 2 to 11 % of juveniles to which parents were not assigned in this study could either be the result from cryptic gene flow from unsampled individuals or rare incursion of immigrants from another swamp via water as well as wind. Triest *et al.* (2014) reported within-lake long-distance dispersal among swamps driven by surface water flow. A combination of dispersal by wind and water increases seed deposition in wetland habitats over long distances (Soomers *et al.* 2013). Extensive short-distance pollen flow promotes the risk of selfing (Whitehead *et al.* 2015). In contrast, we detected relatively long-distance pollen dispersal that also supports the low selfing rate examined.

## Conclusions

In summary, this study provides suggestive evidence for isotropic factor (spatial distance) related FSGS in juveniles and adults of *C. papyrus* in each swamp. Within the swamps, anisotropic FSGS is also associated with an asymmetric wind direction during the dispersal period. Historical gene dispersal inferred from FSGS did not exactly mirror the contemporary pollen and seed dispersal estimated using parentage analysis. Despite the spatial variation in wind direction and strength among swamps, contemporary gene dispersal estimates in terms of seed dispersal distance were short (mean < 55 m) compared to a relatively rare long-distance pollen dispersal (mean 101.2 m). Although an anisotropic pattern of contemporary seed dispersal by wind was detected, we suggest further study to disentangle the relative pollen and seed dispersal patterns in response to long-term wind direction and the effect on FSGS using release-recapture seed dispersal and applying fluorescent dyes for pollen transfer experiments in addition to parentage analysis based on mother plant, the actual seeds and seedlings. These findings suggest that the restoration and conservation of *C. papyrus* populations within the landward edge of the swamps should take account of pollen and seed dispersal distances and heterogeneity in local wind directions during dispersal/pollination periods.

## Sources of Funding

This study was financed by the Vrije Universiteit Brussel—International Relations and Mobility Office (VUB—IRMO) Doctoral Scholarship awarded to A.G. The Doctoral School NSE of the VUB also awarded a travel grant (NSETG-2013-84) to A.G. The BAS 42 funding of the VUB also supported the laboratory analyses of this study. The field work was also partly supported by the small grant, Rufford Foundation (RUF-16029-1).

## Contributions by the Authors

A.G. and L.T. originally formulated the idea. A.G., A.K. and I.S. conceived and designed the field experiments. A.G. and M.G.W. analysed the data. L.T. contributed the reagents and materials. M.G.W. provided the analysis tools and validated the tests. A.G. and A.K. collected plant samples. L.T., I.S. and M.G.W. provided general lab and interpretation advice. A.G. wrote the paper. A.G., M.G.W., A.K., I.S. and L.T. revised the manuscript.

## Conflict of Interest

None declared.

## Supporting Information

The following additional information is available in the online version of this article—

**Table S1**. Locations of the sampling sites, land use types and their attributes.

**Table S2**. The number of seeds released and recaptured during the 6 days dispersal experiment.

**Table S3**. Characteristics of the 15 microsatellite loci for parentage analysis.

**Figure S1**. Field sampling design for genotyping papyrus shoots.

**Figure S2**. Seed dispersal gradient by wind and distribution pattern along the wind directions in C. papyrus swamps (A) Sekelet (SK), (B) Nabega (NB) and (C) Ambo-Bahir (LA) as a function of distance to the source or parent plant. Different symbols represent eight compass directions within 200 m radius from the dispersal point.

**Figure S3**. Cervus parentage assignment analysis. Percentage number of juveniles assigned a parent pair (pp), or a single parent (sp) and not assigned (ns) in each of the papyrus swamps.

**Photo S1**. Lakeside view of papyrus swamps in Ambo-Bahir.

**Photo S2**. Sekelet Giorgis papyrus swamps partly on landside view.

## Supplementary Material

Supporting Information
