# Supplementary material for: Isotropic and anisotropic processes influence fine-scale spatial genetic structure of a keystone tropical plant
Source: AoB Plants. 2018 Jan 6;10(1):plx076. doi: 10.1093/aobpla/plx076 (PMC5777495; doi:10.1093/aobpla/plx076)
Supplement: Supporting Information [file plx076_suppl_supporting_information.doc]

**Table S1 Locations of the sampling sites, land use types and other attributes**

| **Population**  **/swamp** | **Population**  **Acronym** | **Longitude (E)** | **Latitude (N)** | **Altitude (m)** | **Land use types surrounding the wetlands** | **Remarks** |
| --- | --- | --- | --- | --- | --- | --- |
| Nabega | NB | 37°37’10.44” | 11°55’20.35’’ | 1793 | Grasslands  Farm (Agriculture)  Wetland (Minale and Rao, 2012) | Papyrus swamp isolated not connected by water with the other papyrus swamps in the Lake Tana. During the dry season drawdown occurs.  The area of the swamp is about 4.5 ha |
| Ambo-Bahir | LA | 37o19'01.44'' | 11o46'22.26'' | 1790 | Grassland, wetland, crop land, woodland (Hassen and Assen, 2017) | The largest portion of this swamp float on the Lake Tana. Drawdown is common on the margin to the landward. It covers >7.2 ha. Also, hydrologically connected to remotely located swamps fringing the Lake Tana. |
| Sekelet Giorgis | SK | 37o16'13.96'' | 11o48'52.58'' | 1792 | Grassland, forest, woodland, wetland, agriculture field  (Sewnet, 2015) | Protected papyrus swamp seasonally connected to swamps fringing Lake Tana. Drawdown is prominent during the dry season. It covers about 5 ha. |

| **Swamps** | **Number of seeds captured** | **Number of seeds not recovered** | **Number of seeds not dispersed** |
| --- | --- | --- | --- |
| SK | 2510 | 452 | 38 |
| NB | 2420 | 533 | 47 |
| LA | 2371 | 529 | 100 |
| Total | 7301 | 1514 | 185 |

**Table S2** The number of seeds released and recaptured during the six days dispersal experiment.

**Table S3.** Characteristics of the 15 microsatellite loci for parentage analysis. Number of alleles (K), expected heterozygosity (HE), polymorphic information content (PIC), non-exclusion probability of the first parent (NE-1P), non-exclusion probability of the second parent (NE-2P), non-exclusion probability of parent pair (NE-PP) and non-exclusion probability of sib identity (NE-SI).^+^ and ^++^ represent microsatellite developed by Geremew et al. (2017) and Triest et al. (2013), respectively.

| Locus | **Primer sequences (5'-3')** | **Repeat motif** | **Accession No.** | **Allele size** | K | H_E_ | PIC | NE-1P | NE-2P | NE-PP | NE-SI |
| --- | --- | --- | --- | --- | --- | --- | --- | --- | --- | --- | --- |
| Cypap38^+^ | F: 6FAM-AAGGTAATCAATCTGGTCTGCTG  R: CCACTTCTCTTTCTCCTCTCTCAA | (AG)11 | KT873453 | 90-112 | 11 | 0.74 | 0.71 | 0.64 | 0.45 | 0.25 | 0.40 |
| Cypap4^++^ | F:6FAM-AACAAGTTCATTAGTCATGGAGTG  R: TGTTCTCTTGTGGCTCCTGA | (TG) 10 | KC460659 | 138-169 | 12 | 0.84 | 0.82 | 0.49 | 0.32 | 0.14 | 0.35 |
| Cypap14^++^ | F:6FAM-CATGCACATGCTTTTGATGA  R: TGTTCATTGATCGTGCCTTT | (GT)n | KC460665 | 186-200 | 5 | 0.69 | 0.65 | 0.72 | 0.55 | 0.36 | 0.44 |
| Cypap52^+^ | F: 6FAM-CCAAACCCCAACAGAGCAAA  R: ACTTCGGGTGGGATCAAACT | (CCA)9 | KT873455 | 218-246 | 9 | 0.73 | 0.70 | 0.66 | 0.48 | 0.28 | 0.41 |
| Cypap34^+^ | F: VIC-TCATATCACTATATCAGTCTATCAGGG  R: GACACAGGCACACCCAGAA | (AAAGG)8 | KT873452 | 90-118 | 12 | 0.77 | 0.74 | 0.60 | 0.43 | 0.23 | 0.39 |

**Table S3.** Continued

| Locus | **Primer sequences (5'-3')** | **Repeat motif** | **Accession No.** | **Allele size** | K | H_E_ | PIC | NE-1P | NE-2P | NE-PP | NE-SI |
| --- | --- | --- | --- | --- | --- | --- | --- | --- | --- | --- | --- |
| Cypap10^++^ | F: VIC-GACAGCGGCTTGTTTTAAGG  R: TCTCTGCCTTTCACACACTCA | (GT)7 | KC460662 | 143-159 | 9 | 0.52 | 0.51 | 0.854 | 0.69 | 0.51 | 0.56 |
| Cypap13S^++^ | F: NED-CTGTGGCATGGCATCAAAT  R: AAGCACAGGGGTTATGGTTG | (GT)9 (AAT)4 | KC460664 | 163-175 | 6 | 0.58 | 0.53 | 0.820 | 0.66 | 0.49 | 0.52 |
| Cypap3^++^ | F: PET-AAAAGGATTCGATCTGTCACG  R: AAGGGGAAACTTGGTCCTGT | (CT)14(GTGTAA)2 | KC460658 | 154-217 | 11 | 0.62 | 0.56 | 0.788 | 0.64 | 0.47 | 0.49 |
| Cypap28^+^ | F: 6FAM-ACTCACCCACACAGTCACACT  R:TACCAGTGTCGCATCTGCAT | (ACG)9 | KT873451 | 100-120 | 9 | 0.64 | 0.61 | 0.759 | 0.58 | 0.38 | 0.47 |
| Cypap56^+^ | F: 6FAM-GGGGACAATGGCAAAGCTAC  R: TGAACTCTGAAAGACTGAAACCA | (GA)15 | KT873456 | 230-260 | 8 | 0.76 | 0.73 | 0.621 | 0.44 | 0.25 | 0.39 |

**Table S3** Continued

| Locus | **Primer sequences (5'-3')** | **Repeat motif** | **Accession No.** | **Allele size** | K | H_E_ | PIC | NE-1P | NE-2P | NE-PP | NE-SI |
| --- | --- | --- | --- | --- | --- | --- | --- | --- | --- | --- | --- |
| Cypap12^++^ | F: VIC-TGATTTCCTCGCAGCCTAGA  R:AGACCCACAACCCACAAAAA | (TC)8 | KC460663 | 176-182 | 7 | 0.61 | 0.56 | 0.791 | 0.63 | 0.44 | 0.49 |
| Cypap27^+^ | F: VIC-CATGGCTCCCGTGTTAACTT  R: CAAGTATGACTCCAAGCATTTCT | (AT)8 | KT873450 | 82-124 | 9 | 0.62 | 0.59 | 0.769 | 0.58 | 0.37 | 0.49 |
| Cypap23^+^ | F: NED-TGTCCTAATGTTGTTGAATGCTT  R: TTGAACAGATTGGAAGTTTCTTT | (AG)9 | KT873449 | 98-135 | 13 | 0.84 | 0.82 | 0.486 | 0.32 | 0.14 | 0.34 |
| Cypap1^++^ | F: PET-AAGCAGCAAATGAGACAACAA  R: TGTTGGTTGGTTGGTGAAAA | (CAGA)10 | KC460657 | 173-217 | 13 | 0.83 | 0.82 | 0.509 | 0.34 | 0.16 | 0.35 |
| Cypap22^+^ | F: PET-TGGAACTTACAAGCCATACAGATTC  R: CACGGTCAAATGTCTACCAGC | (AAG)14 | KT873448 | 90-115 | 7 | 0.59 | 0.57 | 0.792 | 0.61 | 0.41 | 0.50 |
| Mean |  |  |  |  | 9.4 |  | 0.67 | 0.003 | 2.8x10^-5^ | 1x10^-8^ | 3.7 x 10^-6^ |

**Figure S1.** Schematic representation of sampling design for papyrus shoots for genotyping.


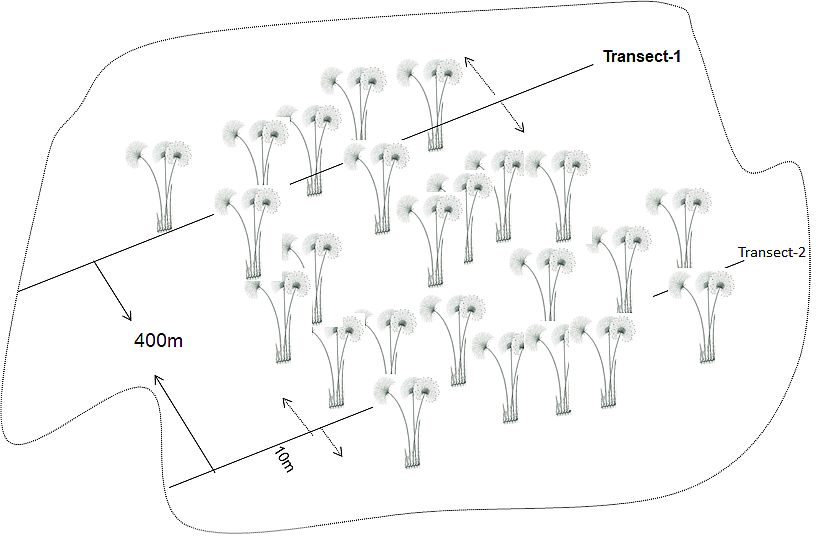


**Figure S2**. Seed dispersal gradient by wind and distribution pattern along the wind directions in *C. papyrus* swamps (a) Sekelet (SK), (b) Nabega (NB) and (c) Ambo-bahir (LA) as a function of distance to the source or parent plant. Different symbols represent eight compass directions within 200m radius from the dispersal point.


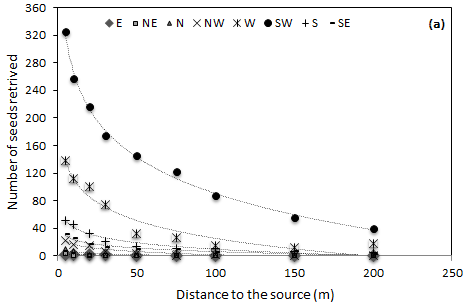


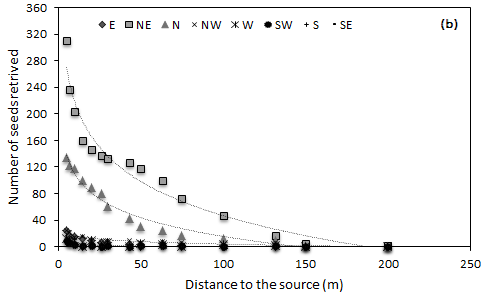


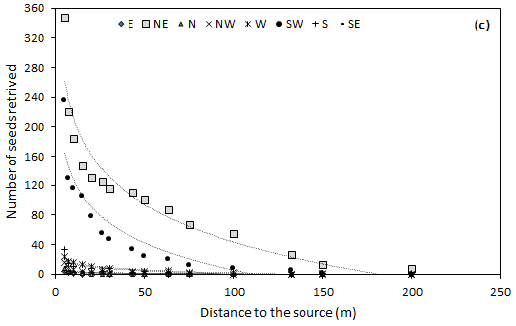


**Fig. S2 continued**

**Figure S3.** Cervus parentage assignment analysis. Percentage number of juveniles assigned a parent pair (pp), or a single parent (sp) and not assigned (ns) in each of the papyrus swamps.


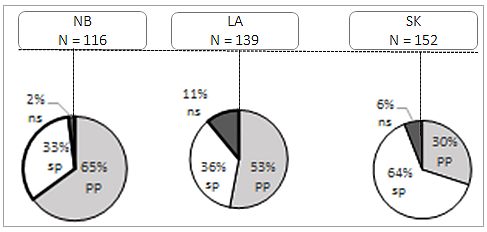


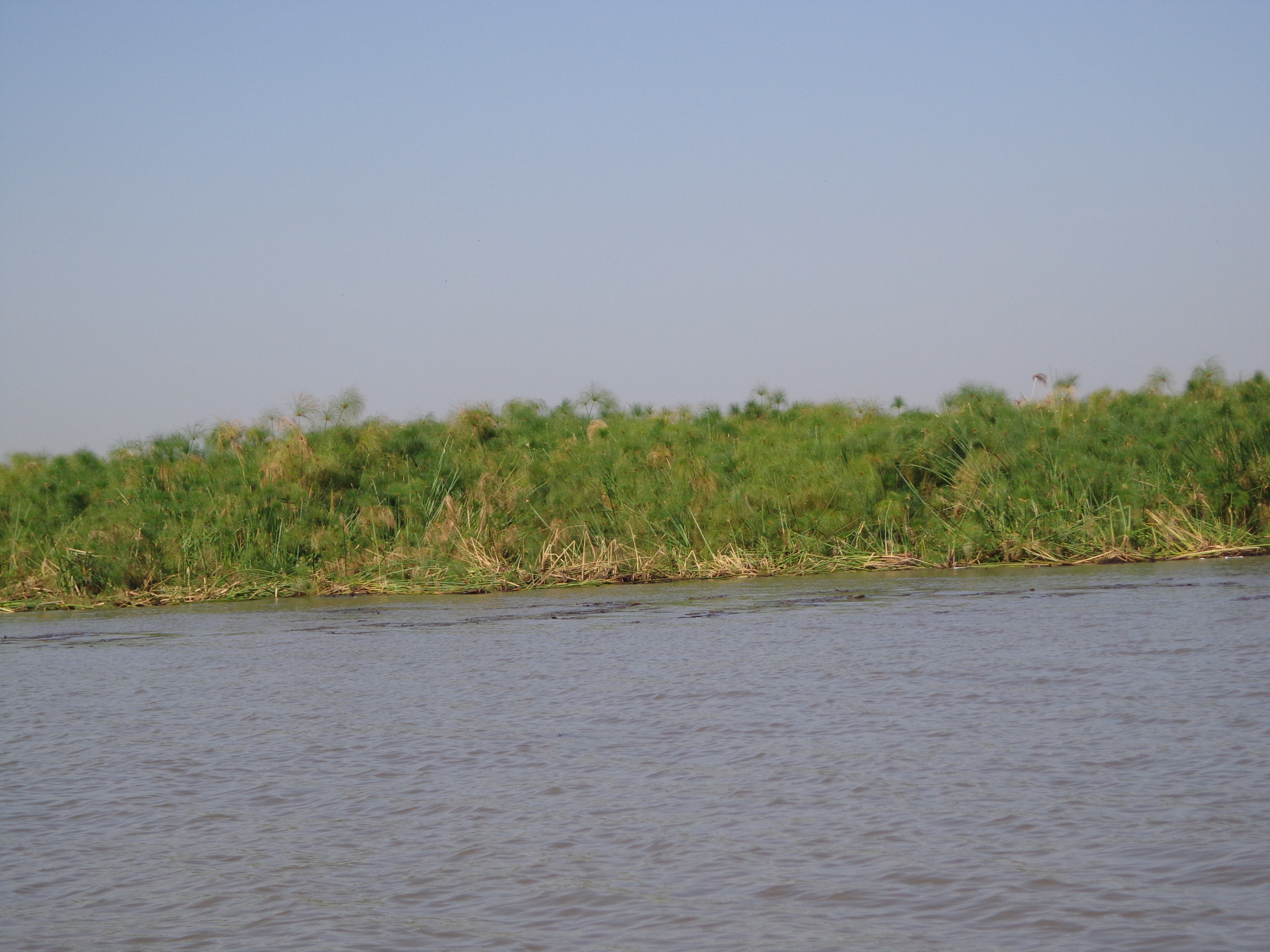


**Photo S1:** Ambobahir papyrus swamp lakeside view

**
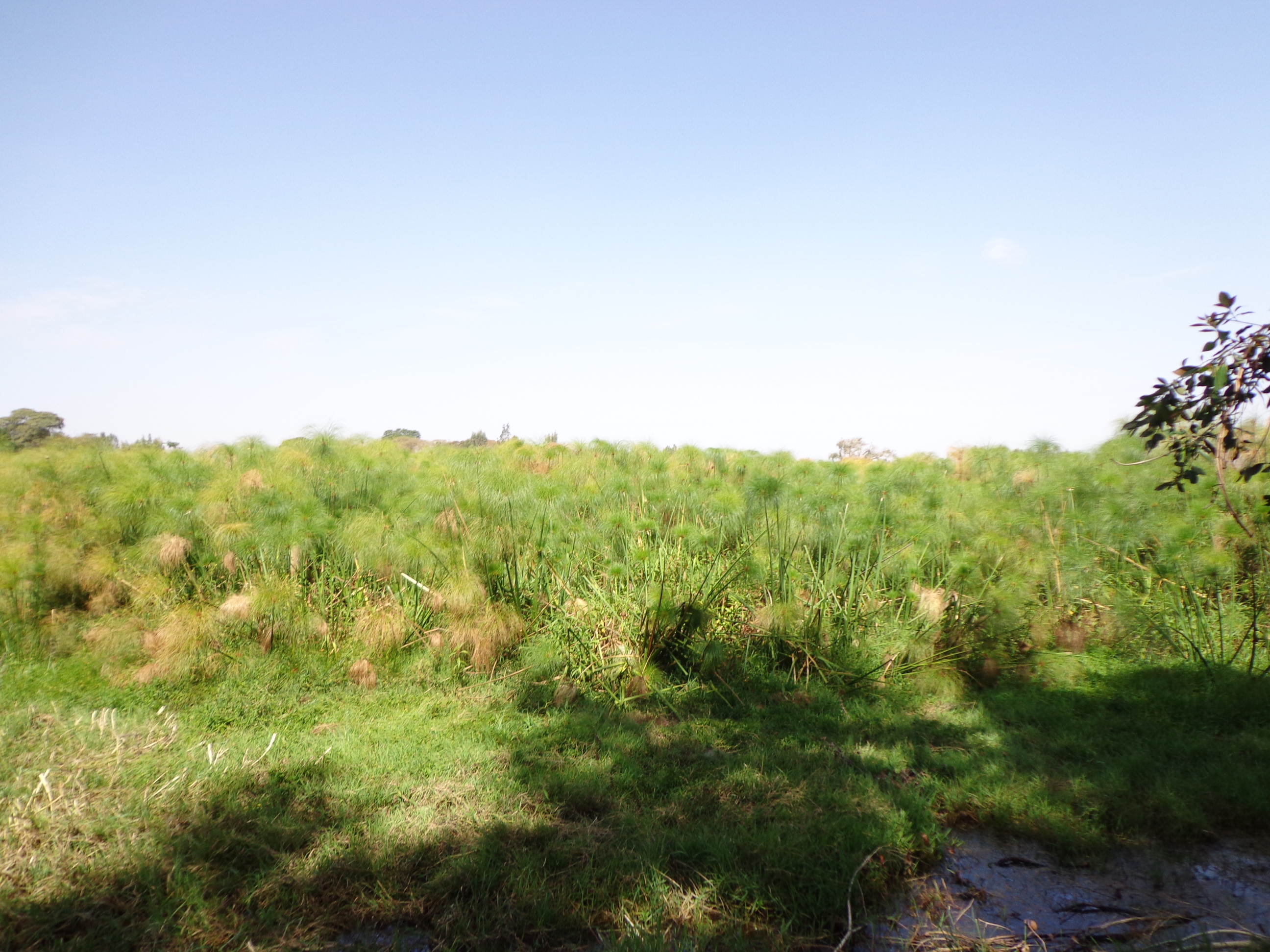
**

**Photos S2.** Sekelet papyrus swamp partly land side view
